# Structural brain changes in the anterior cingulate cortex of major depressive disorder individuals with suicidal ideation: Evidence from the REST-meta-MDD project

**DOI:** 10.1017/S0033291724003283

**Published:** 2025-02-07

**Authors:** Zhiqiang Yi, Luyao Xia, Junfei Yi, Yanfei Jia, Luhua Wei, Shengli Shen, Nan Wu, Dongmei Wang, Huixia Zhou, Xingxing Li, Chao-Gan Yan, Xiang-Yang Zhang

**Affiliations:** 1 Department of Neurosurgery, Peking University First Hospital, Beijing, China; 2 Department of Psychology, Teachers’ college of Beijing Union University, Beijing, China; 3 Learning and Psychological Development Institution for Children and Adolescents, Beijing Union University, Beijing, China; 4 Neurology Department, Peking University First Hospital, Beijing, China; 5 Institute of Psychology, Chinese Academy of Sciences, Beijing, China; 6 Department of Psychology, University of Chinese Academy of Sciences, Beijing, China; 7 International Big-Data Center for Depression Research, Institute of Psychology, Chinese Academy of Sciences, Beijing, China; 8 Magnetic Resonance Imaging Research Center and Research Center for Lifespan Development of Mind and Brain (CLIMB), Institute of Psychology, Chinese Academy of Sciences, Beijing, China; 9 Anhui Mental Health Center, Hefei Fourth People’s Hospital, Affiliated Mental Health Center of Anhui Medical University, Hefei, China

**Keywords:** anterior cingulate cortex, gray matter volume, magnetic resonance imaging, major depressive disorder, suicidal ideation

## Abstract

**Background:**

Suicidal ideation (SI) is very common in patients with major depressive disorder (MDD). However, its neural mechanisms remain unclear. The anterior cingulate cortex (ACC) region may be associated with SI in MDD patients. This study aimed to elucidate the neural mechanisms of SI in MDD patients by analyzing changes in gray matter volume (GMV) in brain structures in the ACC region, which has not been adequately studied to date.

**Methods:**

According to the REST-meta-MDD project, this study subjects consisted of 235 healthy controls and 246 MDD patients, including 123 MDD patients with and 123 without SI, and their structural magnetic resonance imaging data were analyzed. The 17-item Hamilton Depression Rating Scale (HAMD) was used to assess depressive symptoms. Correlation analysis and logistic regression analysis were used to determine whether there was a correlation between GMV of ACC and SI in MDD patients.

**Results:**

MDD patients with SI had higher HAMD scores and greater GMV in bilateral ACC compared to MDD patients without SI (all p < 0.001). GMV of bilateral ACC was positively correlated with SI in MDD patients and entered the regression equation in the subsequent logistic regression analysis.

**Conclusions:**

Our findings suggest that GMV of ACC may be associated with SI in patients with MDD and is a sensitive biomarker of SI.

## Introduction

Major depressive disorder (MDD) is a psychiatric disorder clinically characterized by changes in mood and cognition, loss of interest or pleasure, lasting at least 2 weeks, and sometimes accompanied by suicidal behavior (Dong et al., 2021; Zhdanava et al., 2021). From 2001 to 2020, the lifetime prevalence of MDD is 19% (95% CI: 0.12–0.26) globally, with a pooled 1-year prevalence of about 8% (95% CI: 0.05–0.12) (Shorey, Ng, & Wong, 2022). In mainland China, the lifetime prevalence and 12-month prevalence of MDD were 3.4% (95% CI: 2.9–3.9) and 2.1% (95% CI: 1.8–2.4), respectively (Huang et al., 2019). The incidence of suicidal ideation (SI) in Chinese individuals with MDD is as high as 53.1% (95% CI: 42.4–63.4%) (Dong et al., 2018). Therefore, there is a need to search for risk factors associated with SI in MDD patients, and to provide regular screening and effective intervention for suicidal behavior (Dong et al., 2018; Dong et al., 2021). SI in MDD patients is significantly associated with many sociodemographic characteristics, such as lower educational attainment, Caucasian or African American, male sex, unemployment, and psychiatric treatment, as well as clinical characteristics, such as previous suicide attempts, younger age of onset, and glucose disturbances (Dong et al., 2021; Sokero et al., 2003; Trivedi et al., 2013). However, the underlying neural mechanisms behind these risk factors remain unclear.

Various cortical and limbic structures, including the anterior cingulate cortex (ACC), have been implicated in MDD (Chen et al., 2018; Ibrahim, Kulikova, Ly, Rush, & Sherwood Brown, 2022; Lemke et al., 2022; Sindermann et al., 2021). The ACC is a limbic structure that plays an important role in regulating emotion, attention, motivation, and information processing (Bliss-Moreau, Santistevan, Bennett, Moadab, & Amaral, 2021; Hu et al., 2021; Li et al., 2021; Smith, Asada, & Malenka, 2021). It is part of the emotional circuit known as the “anterior compartment.” This circuit also includes the amygdala and the anterior insula, which are anatomically interconnected and metabolically integrated (Ibrahim et al., 2022; Mayberg et al., 1999). Multiple magnetic resonance imaging (MRI) studies have reported reduced ACC volume in MDD patients (Belleau, Treadway, & Pizzagalli, 2019; Mertse et al., 2022; Riva-Posse, Holtzheimer, & Mayberg, 2019). This finding was confirmed by a larger study in which individuals with higher self-reported severity of depressive symptoms had reduced right-sided ACC volume, an association that was only significant in men (Ibrahim et al., 2022).

In patients with MDD, ACC dysfunction is associated with SI (Lewis et al., 2020). Reduced gray matter volume (GMV) of the ACC has been found in suicide attempters among individuals with various psychiatric disorders (Bani-Fatemi et al., 2018). GMV of ACC may be a sensitive biomarker of SI risk in MDD patients. Abnormalities in brain structure may be associated with the development and progression of SI in MDD patients. However, the expression of GMV in the ACC of MDD patients with SI remains unclear.

A large number of previous studies have attempted to reveal the neural mechanisms behind suicidal behaviors through structural neuroimaging techniques (Chen et al., 2024; Guo et al., 2023). Previous studies have shown an association between changes in GMV in the brains and SI in patients with MDD (Chen et al., 2024; Guo et al., 2023). Reduced GMV in the bilateral caudate nucleus (Ho et al., 2021), right nucleus accumbens (Ho et al., 2021), dorsal striatum (Ho et al., 2018), lingual gyrus (Wang et al., 2021), right insula (Ge et al., 2021), bilateral dorsolateral prefrontal cortex (DLPFC; R Zhang et al., 2020), and right ventrolateral prefrontal cortex (R Zhang, Wei, et al., 2020) was associated with SI in patients with MDD. Two comprehensive meta-analyses reported significant changes in cortical and subcortical structure in MDD patients with suicidal tendencies compared to healthy controls, including smaller total intracranial and subcortical volumes, larger ventricular volumes, smaller thalamus and globus pallidus volumes, and smaller inferior parietal lobe surface area (Campos et al., 2021; Renteria et al., 2017). These findings suggest that neurobiological markers are associated with suicidal behavior in depressed patients (Chen et al., 2024; Guo et al., 2023). However, no diagnostic clinical imaging markers and predictors have been identified (Yan et al., 2019). One of the reasons for these contradictory results is that small sample studies have low statistical power and are prone to false positive results (Button et al., 2013; Poldrack et al., 2017). Second, different workflows can lead to differences in the results of MRI datasets (Botvinik-Nezer et al., 2020). Finally, physiological confounders such as head motion can also affect results (Ciric et al., 2018). The REST-meta-MDD project shared 25 research cohorts, including R-fMRI data from 1,300 MDD patients and 1,128 healthy control participants (Chen et al., 2022). A standardized preprocessing procedure was used for each subject to minimize heterogeneity in preprocessing methods (Chen et al., 2022; Yan, Wang, Zuo, & Zang, 2016). All data were corrected for head movements using the Friston-24 model as the default setting (Yan et al., 2013). The project improved statistical power by pooling data across centers while minimizing the impact of heterogeneous analysis strategies (Chen et al., 2022).

However, there are no large, multicenter studies investigating structural brain changes in MDD patients with SI by GMV. In particular, no study has used samples from the Chinese REST-meta-MDD project (Yan et al., 2019), which addresses the problems of limited statistical power and analytical heterogeneity of small samples. In this study, we sought to elucidate the neurological basis of MDD patients with SI by comparing the GMV of structural MRI of ACC brain regions in MDD patients with and without SI from the REST-meta-MDD project. We further sought to determine whether these altered brain regions were risk factors for SI in MDD patients. To the best of our knowledge, similar studies have not been conducted in MDD patients in the Chinese population. We hypothesized that there is a difference in GMV of ACC between MDD patients with and without SI.

## Methods

### Participants

Participants in this study included 1,300 individuals diagnosed with MDD and 1,128 healthy controls. As part of the REST-meta-MDD consortium, all participants were recruited from 25 Chinese consortium members in 18 hospitals based on similar inclusion and exclusion criteria (Liu et al., 2021; Long et al., 2020; Yan et al., 2019). All individuals provided written informed consent before participating in the study. The original studies were approved by the local Institutional Review Boards, and then by the Institutional Review Board of the Institute of Psychology, Chinese Academy of Sciences for sharing deidentified and anonymized data. Consortium members provided only basic information, including diagnosis, duration of illness, sex, age, education status, and the 17-item Hamilton Depression Rating Scale (HAMD).

A total of 88 MDD patients and 38 healthy controls were excluded from the analysis. Patient inclusion criteria were as follows: (1) age over 18 and under 65 years; (2) years of education greater than 5 years; (3) meeting the criteria for MDD based on the Structured Clinical Interview for the Diagnostic and Statistical Manual of Mental Disorders-IV (DSM-IV) or International Classification of Diseases 10 (ICD-10); and (4) having a total score of no less than 8 on the 17-item HAMD at the time of scanning.

The exclusion criteria were as follows: (1) exclusion of patients with late-onset depression and patients in remission; (2) exclusion of subjects who lacked basic information, such as incomplete information on gender, age, and education; (3) exclusion of subjects with poor quality imaging data, including poor spatial normalization; (4) exclusion of subjects with group mask coverage of less than 90% or with a head-averaged framewise displacement of greater than 0.2 mm; and (5) exclusion of subjects from research sites with sample sizes less than 10 people (Yan et al., 2019).

We matched individuals with and without SI for age and sex. MDD patients were also matched with healthy controls for age and sex. Therefore, this study included 235 healthy controls and 246 MDD patients, including 123 MDD patients with and without SI each.

### Clinical measures

The 17-item HAMD was used to assess the level of depression in MDD patients (Hamilton, 1960). The HAMD scale consists of 17 items, 8 of which are on a 5-point level (0: absent, 4: severe) and 9 items are on a 3-point level (0: absent, 2: severe). The presence and degree of depression is determined by the total score of HAMD. The Chinese version of this scale has been shown to have good reliability and validity (Dong et al., 2021; Lin, 1986; Sun, Li, Yu, & Li, 2017).

SI was measured by HAMD item 3 (suicide). The item has the following alternative statements: 0 = not present, 1 = feeling that life is not worth living, 2 = wishing for or repeatedly thinking about death, 3 = having suicidal thoughts, and 4 = attempting suicide. For the purposes of this study, we defined someone had SI via a score of ≥3 on HAMD item 3. Specifically, if an individual scores 3 or 4 on HAMD item 3, he/she will be classified into the group with SI, and individuals with scores of 0, 1, and 2 will be classified into the group without SI (Ge, Jiang, Wang, Yuan, & Zhang, 2020; Li et al., 2019; Vuorilehto et al., 2014).

### Image acquisition, preprocessing, and quality control

Structural T1-weighted MRI brain scans were performed at each hospital (Yan et al., 2019). Images were preprocessed using DPARSF software, and image processing was performed using SPM 8 and VBM 8 toolboxes (http://dbm.neuro.unijena.de/vb) (Chao-Gan & Yu-Feng, 2010; Yan et al., 2016). T1 images were normalized using template space, and then segmented into gray matter, white matter (WM), and cerebrospinal fluid. Individual native spaces were then converted to MN1 space using the Diffeomorphic Anatomical Registration Through Exponential Lie algebraic tool (Ashburner, 2007). Once preprocessing was complete, quality checks were performed using the two modules called “Display one slice for all images” and “Check sample homogeneity using covariance.” The normalized images were smoothed using an 8 mm full width at half maximum Gaussian kernel.

### Statistical analysis

Demographic and clinical variables were compared using the χ^2^ test, categorical variables using Fisher’s exact test, and continuous variables using analysis of variance. Normal distribution was assessed using the Kolmogorov–Smirnov test (p > 0.05). The Levene’s test was used to assess homogeneity of variance (p > 0.05), and the Mauchly’s test was used for the assumption of sphericity (p > 0.05).

Using the DPABI toolbox version 7.0 (Yan et al., 2016), GMV in the ACC was compared and analyzed using two-sample *t* tests with age, education, HAMD, and head movement as covariates to determine differences between MDD patients and healthy controls. In the patient group, two-sample *t* tests were performed with concomitant SI as the independent variable, GMV in ACC as the dependent variable, and age, education, first episode, medication status, disease duration, HAMD, and head movement as covariates. The mask was areas of interest for ACC defined by the Harvard-Oxford atlas (Carlson et al., 2022; Desikan et al., 2006; Frazier et al., 2005). In addition, we adjusted multiple testing using 1000 permutations with threshold-free cluster enhancement (TFCE) correction.

Similarly, we performed a correlation analysis between GMV in the ACC and demographic and as well as clinical variables. Brain regions were considered significant according to the Gaussian random field theory (GRF) correction (cluster-P <0.01, voxel-P <0.001). We then extracted GMV under the clusters with significant correlations and used multiple regression models to analyze the factors influencing abnormal GMV in the ACC of MDD patients with and without SI.

All statistical analyses were calculated using R version 4.3.1 (http://cran.r-project.org). Data are presented as mean ± standard deviation. The *p*-value was set at a two-tailed significance level ≤0.05.

## Results

### Demographic and clinical characteristics

Demographic information is shown in Table 1. There was a significant difference in education between MDD patients and healthy controls (p < 0.001). There were also significant differences in HAMD (p < 0.001) and head motion (p < 0.05) between MDD patients with and without SI. These factors were controlled as covariates in subsequent analyses.

**Table 1.** Demographics of MDD patients and healthy controls

	MDD		Healthy controls (n = 235)	Suicidal ideation F/χ^2^ (p value)	Diagnose F/χ^2^ (p value)
	With SI (n = 123)	Without SI (n = 123)			
Sex (M/F)	39/84	39/84	78/157	0 (1.000)	0.063 (0.803)
Age (years)	31.12 ± 10.08	31.61 ± 9.65	32.63 ± 10.56	0.15 (0.699)	1.857 (0.174)
Education (years)	12.07 ± 3.46	12.33 ± 3.51	13.84 ± 3.70	0.343 (0.559)	25.17 (<0.001)
First episode (yes)	57	47		1.349 (0.245)	
On medication (yes)	29	32		0.087 (0.768)	
Disease duration (months)	23.60 ± 40.89	18.06 ± 48.83		0.931(0.336)	
HAMD	25.98 ± 6.24	23.04 ± 5.61		15.14 (<0.001)	
Head motion (mean FD_Jenkinson)	0.07 ± 0.04	0.08 ± 0.04	0.08 ± 0.04	6.129 (<0.05)	0.108 (0.743)

*Note:* Mean ± SD; HAMD: Hamilton Depression Scale; MDD: major depressive disorder; SI: suicidal ideation.

### Differences in GMV in the ACC of MDD patients with and without SI and healthy controls

As shown in Supplementary Figure S1, GMV of the right ACC was significantly smaller in MDD patients than in healthy controls (cluster 1: peak voxel *X* = 6, *Y* = 18, *Z* = 21; *t* = 4.387; cluster 2: peak voxel *X* = 6, *Y* = 15, *Z* = 22.5; *t* = 6.201). After adding age and education as covariates, one of the differences remained significant (cluster size = 104; peak voxel *X* = 6, *Y* = 15, *Z* = 22.5; *t* = 6.131). All of these results passed 1000 permutations with TFCE correction.

**Figure 1.**
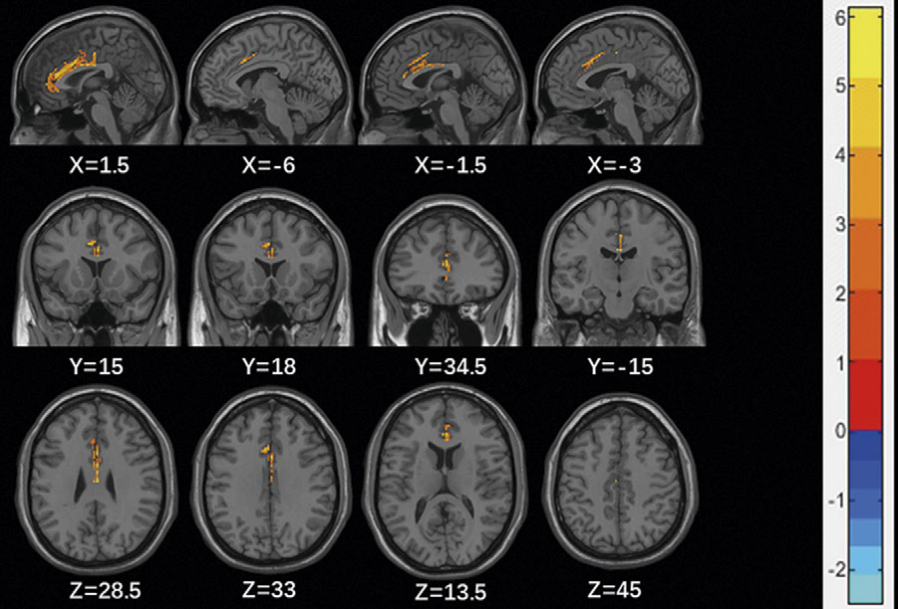
Clusters with significant differences in GMV in the ACC between MDD patients with SI and without SI based on two-sample *t* tests. Red and blue colors denote increased and decreased GMV. The color bars indicate the T-value (1000 permutations with TFCE correction).

Furthermore, we analyzed the differences in GMV of ACC between MDD patients with and without SI. The GMV of bilateral ACC was greater in MDD patients with SI compared to MDD patients without SI (see Table 2 and Figure 1). After adding age, education, first episode, medication status, disease duration, HAMD and head motion as covariates, significant differences remained between the four clusters (see Supplementary Table S1). All of these results passed 1000 permutations with TFCE correction.

**Table 2.** Regions showing significant differences between GMV of MDD patients with SI and without SI

Brain areas	Cluster size	BA	L/R	MNI coordinate of peak voxel			T-value (peak)
				x	y	z	
Cingulate gyrus, anterior division	653	24	R	1.5	15	28.5	6.154
Cingulate gyrus, anterior division	270	24	L	–6	18	33	5.322
Cingulate gyrus, anterior division	14	24	L	−1.5	34.5	13.5	4.208
Cingulate gyrus, anterior division	3	23	L	−3	−15	45	4.257

Note: BA = Brodmann area; MNI coordinates = Coordinates of primary peak locations in the Montreal Neurological Institute space; T-statistical value of peak voxel showing GMV differences between groups; 1000 permutations with TFCE corrected for multiple comparisons across space.

### Differences in the relationships between ACC GMV and demographic characteristics in MDD patients with and without SI and healthy controls

**Figure 2.**
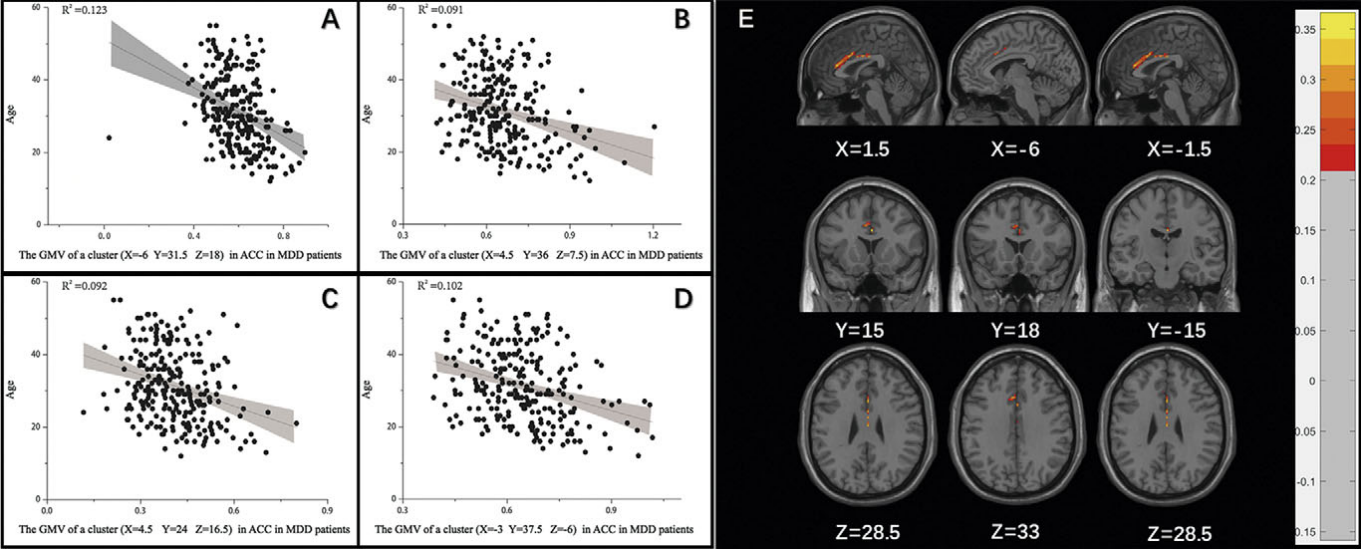
Correlation between GMV in the ACC and age as well as SI in MDD patients A: Significant negative correlations of a cluster (X = -6 Y = 31.5 Z = 18) in ACC and age (p < 0.05). B: Significant negative correlations of a cluster (X = 4.5 Y = 36 Z = 7.5) in ACC and age (p < 0.05). C: Significant negative correlations of a cluster (X = 4.5 Y = 24 Z = 16.5) in ACC and age (p < 0.05). D: Significant negative correlations of a cluster (X = -3 Y = 37.5 Z = -6) in ACC and age (p < 0.05). E: Clusters with significant correlation between GMV in the ACC and SI in MDD patients. The color bar represents the correlation coefficient *r* (adjusted by GRF, cluster-P <0.05, voxel-P <0.001).

Figure 2 shows that ACC GMV was significantly correlated with age and SI in MDD patients. The specific data are presented in Supplementary Table S2. However, no correlation was found between GMV of ACC and demographic characteristics in healthy controls (all p > 0.05).

A negative correlation between GMV in right ACC and sex was observed in MDD patients with SI (cluster size = 42, peak voxel X = 1.5, Y = −6, Z = 36; r = −0.400, adjusted by GRF, cluster-P <0.05, voxel-P <0.001).

Table 3 shows the relationship between GMV of ACC and demographic characteristics in MDD patients without SI. A negative correlation between GMV of left ACC and age (Supplementary Figure S2) and between GMV of bilateral ACC and sex was noted in MDD patients without SI (two p < 0.05).

**Table 3.** Associations between the GMV in the ACC and demographic characteristics in MDD patients without SI

Variables	Brain areas	Cluster size	BA	L/R	MNI coordinate			r	p
					x	y	z		
age	Cingulate gyrus, anterior division	32	24	L	−6	36	16.5	−0.363	<0.05
sex	Cingulate gyrus, anterior division	878	24	L	−7.5	12	33	−0.533	<0.05
	Cingulate gyrus, anterior division	463	32	R	10.5	30	22.5	−0.547	<0.05
	Cingulate gyrus, anterior division	315	23	R	7.5	−15	42	−0.462	<0.05

*Note:* BA = Brodmann area; MNI coordinates = coordinates of primary peak locations in the Montreal Neurological Institute space; *p*-value for Pearson correlation; adjusted by GRF, cluster-P <0.05, voxel-P <0.001.

Further multiple regression analysis showed that in MDD patients, sex (t = −5.287, p < 0.001), age (t = −5.731, p < 0.001), and first episode (t = 2.557, p < 0.05) were factors associated with age-related GMV of the first cluster. Sex (t = −5.203, p < 0.001), age (t = −5.107, p < 0.001), first episode (t = 2.551, p < 0.05), and on medication (t = 1.978, p < 0.05) factors associated with age-related GMV of the second cluster. Sex (t = −3.248, p < 0.01), age (t = −5.284, p < 0.001), and education (t = −2.361, p < 0.05) were factors associated with age-related GMV of the third cluster. Sex (t = −5.424, p < 0.01), age (t = −5.12, p < 0.001), and first episode (t = 2.866, p < 0.05) were factors associated with age-related GMV of the fourth cluster.

Multiple regression analysis showed that among MDD patients with SI, sex (t = −5.078, p < 0.001), age (t = −2.350, p < 0.05), and education (t = −2.293, p < 0.05) were factors associated with sex-related GMV of the cluster.

In MDD patients without SI, sex (t = −5.636, p < 0.001), age (t = −5.4, p < 0.001), and first episode (t = 2.611, p < 0.05) were factors associated with age-related GMV of the cluster. Sex (t = −8.413, p < 0.001) and age (t = −3.621, p < 0.001) were factors associated with sex-related GMV of the first cluster. Sex (t = −7.622, p < 0.01), age (t = −2.355, p < 0.05), and first episode (t = 2.280, p < 0.05) were factors associated with sex-related GMV of the second cluster. Sex (t = −7.306, p < 0.001) and age (t = −2.693, p < 0.001) were factors associated with sex-related GMV of the third cluster.

In addition, in MDD patients, logistic regression was performed with the presence or absence of comorbid SI as the dependent variable. In patients with MDD, HAMD (t = 4.83, p < 0.001), medication (t = −2.593, p < 0.001), and GMV of cluster were significantly associated with SI (t = 4.418, p < 0.001; t = 2.934, p < 0.01).

## Discussion

This study had three main findings as follows: (1) MDD patients with SI scored higher on HAMD compared to MDD patients without SI, (2) GMV in the ACC region was significantly smaller in MDD patients compared to healthy controls. MDD patients with SI had greater GMV of bilateral ACC compared to MDD patients without SI, and (3) GMV of bilateral ACC was positively correlated with SI in MDD patients and entered the regression equation in the subsequent logistic regression analysis. Thus, we found that there was a significant difference in GMV of ACC between MDD patients with and without SI, and that GMV of ACC was associated with SI in MDD patients.

Our study found that MDD patients with SI had higher HAMD score compared to MDD patients without SI, suggesting that MDD patients with SI may have more severe depressive symptoms, which is consistent with previous findings (Cai et al., 2021; Guo et al., 2023; Khandoker et al., 2017).

As the first step in suicidal behavior, SI is the product of the interaction of multiple risk factors, such as genetic and environmental factors (Dada et al., 2021; Ropaj, 2023). Most psychiatric disorders increase suicide risk, especially depression (Cai et al., 2021; Dada et al., 2021; Dai et al., 2022; Nassan et al., 2022; Schneider, Chen, Lungu, & Grasso, 2020). In patients with MDD, a higher prevalence of SI tends to be associated with more severe depressive symptoms (Cai et al., 2021). A variety of treatments, including electroconvulsive therapy, repetitive transcranial magnetic stimulation (rTMS), and medications, can simultaneously alleviate patients’ depressive symptoms and reduce their SI (Hetrick et al., 2021; Hochschild et al., 2022; Ionescu et al., 2021; Li, Yu, et al., 2021; Pan et al., 2020). Serum cystatin C (Cys C) exerts biological functions in several aspects including bioactivity and neurophysiology, and it may affect the risk of depression in a number of ways (Daria et al., 2020; Islam et al., 2020; Zi & Xu, 2018). Changes in Cys C levels may be associated with increased neuronal inflammation, which may increase some of the inflammatory factors involved in suicidal tendency in depressed patients (Sun, Chen, & Li, 2021). For example, IL-6 and TNF-α affect suicide risk in depressed patients by affecting the serotonergic system (Brundin, Bryleva, & Thirtamara Rajamani, 2017; Pandey et al., 2012). Cys C may also play an important role in suicide risk in depressed patients by inducing neuronal apoptosis and disrupting WM and brain amyloid deposition (Jia et al., 2010; Mitaki et al., 2011; Sun et al., 2021). Furthermore, traumatic experiences are often associated with more severe depression, which in turn may lead to more generalized SI (Cai et al., 2021). Although depressive symptoms in MDD patients are closely associated with SI, they remain independent of each other. Individuals with SI have higher levels of anhedonia than those without SI, but depression does not explain the association between high levels of anhedonia and SI (Ducasse et al., 2018). Therefore, the relationship between depressive symptoms and SI needs further research.

The present study demonstrated that the GMV in the ACC region was significantly smaller in MDD patients compared to healthy controls. Consistent with previous findings, MDD patients exhibited ACC atrophy compared to healthy controls (He et al., 2022). This atrophy may be associated with MDD, a degenerative mental disorder (Schmaal et al., 2020; Stein et al., 2021; Zhang, Wei, et al., 2020). The ACC is a key region associated with MDD (Cole et al., 2020; Crowell et al., 2019; Rappaport, Kandala, Luby, & Barch, 2020). In a study using rTMS to treat MDD, the left ACC of the pathological neural network was found to be connected to the DLPFC, and the abnormalities of the frontal and parietal lobes were improved after treatment (Belleau et al., 2019; Philip et al., 2018).

However, in this study, the GMV of the bilateral ACC was greater in MDD patients with SI compared to MDD patients without SI, suggesting that there is a significant difference in the GMV of the ACC between MDD patients with and without SI, as evidenced by the significantly lower atrophy of the bilateral ACC of MDD patients with SI. This is consistent with previous findings in MDD patients with higher rates of suicide attempts and type I bipolar disorder (Duarte et al., 2017; Rizk et al., 2019). The ACC plays an important role in cognitive reappraisal of negative emotions and serves the attentional control network (Buhle et al., 2014; Shenhav, Cohen, & Botvinick, 2016). Previous studies have shown that a subset of suicide attempters with higher lethality have higher levels of pre-suicide planning and lower levels of delay discounting, so that they can suppress their desire for immediate gratification and choose delayed rewards (Chaudhury et al., 2016; Dombrovski et al., 2011). Suicide attempters with higher lethality also perform better on an object alternation task compared to those with lower lethality, suggesting greater executive control and response organization (Keilp et al., 2014). Reduced globus pallidus volume has also been found in suicide attempters with a strong desire to die and in those with higher lethality rates, which is associated with their non-impulsive temperament (Dombrovski et al., 2012; Vang, Ryding, Traskman-Bendz, van Westen, & Lindstrom, 2010). MDD patients with SI have greater GMV in the ACC, which may be associated with their better cognitive control and overlap with suicide attempters, with higher mortality due to suicidal behaviors, representing a non-impulsive subgroup (Rizk et al., 2019). At the same time, increased GMV may represent a compensatory process for the prolonged high stress prior to the onset of suicidal behavior (Rizk et al., 2019). Increased microglial density in suicidal individuals suggests that microglial activation may be a result of pre-suicidal stress (Steiner et al., 2008). This microglia activation leads to increased somatic cell size and coarsening of branching processes, a change that may be reflected in the larger GMV observed in the current study (LaVoie, Card, & Hastings, 2004; Rizk et al., 2019). Finally, heterogeneity in suicidal behavior, especially in terms of lethality and intent, may contribute to inconsistent results (Rizk et al., 2019). Suicidal behaviors are diverse, ranging from highly lethal suicide attempts to less lethal ones; from impulsive suicidal behaviors to well-planned and determined suicidal behaviors (Chaudhury et al., 2016; Keilp et al., 2014; Rizk et al., 2019). Overrepresentation of one type of study may produce different results than other studies (Rizk et al., 2019). Individuals with highly lethal suicide attempts may be similar to suicidal individuals in terms of demographics, clinical presentation, and brain biology (Chaudhury et al., 2016; Rizk et al., 2019). Our results are similar to those of another study of MDD patients with a history of at least one highly lethal suicide attempt who were not receiving medication, suggesting that GMV is greater in patients with SI (Rizk et al., 2019). However, the physiological mechanisms underlying increased GMV remain to be further investigated.

In this study, GMV in bilateral ACC was positively correlated with SI in MDD patients. Subsequent regression analysis showed that the GMV of cluster significantly associated with SI in MDD patients was related to SI in MDD patients. The ACC is a structure in the medial prefrontal cortex consisting of multiple functional divisions (Palomero-Gallagher et al., 2019). The ACC is involved in emotion regulation, thought-emotion valence, decision-making, and impulsivity, and is central to neuroanatomical models of depression (Gradone et al., 2023; van Heeringen, Bijttebier, Desmyter, Vervaet, & Baeken, 2014; Wise et al., 2017). There is accumulating evidence that ACC abnormalities are associated with suicidality across different diagnostic categories of psychiatric disorders, including MDD (He et al., 2022; Wagner et al., 2011), schizophrenia (van Heeringen et al., 2014), and borderline personality disorder (Duarte et al., 2017). This transdiagnostic perspective is supported by recent studies that show the consistency in certain brain networks despite current psychopathology (Goodkind et al., 2015; Gradone et al., 2023; Wise et al., 2017). In this study, SI increased with increasing GMV in the ACC of MDD patients. One possible explanation is that overactivity of the ACC may lead to increased brain plasticity and thus become larger (Fears et al., 2015; Lisy et al., 2011). This could act as a compensatory mechanism for regulating emotional states, mitigating failures in frontal lobe “top-down” regulation (Duarte et al., 2017). Furthermore, epigenetic dysregulation of glucocorticoid receptors and serotonin receptor binding plays an important role in this process (Bartlett et al., 2022; Pantazatos et al., 2022; Rizavi et al., 2023). Taken together, ACC may be involved in the pathological mechanism of SI in MDD patients, suggesting that GMV in the ACC may be a biomarker for SI in sensitive MDD populations.

In contrast to our findings, previous studies have shown that the GMV of ACC is smaller in MDD patients with suicide attempts compared to MDD patients without suicide attempts and healthy controls, and that the GMV of ACC progressively decreases with increasing SI in MDD patients (He et al., 2022; Wagner et al., 2011). Differences from the current study may be due to heterogeneity in the degree of suicide attempts and suicide fatalities, as well as differences in the way SI subgroups were distinguished (Klonsky, May, & Saffer, 2016). Similarly, a previous study used only 30 individuals recruited from the inpatient and outpatient services of the Department of Psychiatry and Psychotherapy at Friedrich-Schiller University (Wagner et al., 2011). Only 129 MDD patients were used in another study with Han Chinese (He et al., 2022). It is different from the large sample size of Chinese Han individuals in this study. In addition, the use of different methods such as Freesurfer and VBM in earlier versions of the data processing software SPM2 may have led to differences in the results (He et al., 2022; Wagner et al., 2011).

There is evidence that a long disease duration is associated with a poor prognosis in MDD patients (Altamura, Serati, & Buoli, 2015). Long disease duration impairs the immune system and central nervous system (Altamura et al., 2015; Arango, Breier, McMahon, Carpenter, & Buchanan, 2003). This process is associated with glutamate-mediated neuronal cytotoxicity and activation of the hypothalamic–pituitary–adrenal (HPA) axis (Matsuo et al., 2010). This biological abnormality may be the result of a neurodegenerative model (Altamura, Buoli, & Pozzoli, 2014; Skeppar et al., 2013). Atypical antipsychotics and antidepressants with manic and mood-stabilizing effects can block the neurodegenerative process associated with long disease duration, especially during the first year of illness in patients with first episode MDD (Andreasen, 2010; Ho, Andreasen, Ziebell, Pierson, & Magnotta, 2011; van Haren et al., 2011). Long disease duration and low medication adherence may contribute to an increased risk of SI in patients with MDD (Altamura et al., 2015). Antidepressants are first-line medications for the treatment of severe MDD patients and have been shown to improve dysfunction and alter neural activation and brain structure (Cleare et al., 2015; Dichter, Gibbs, & Smoski, 2015). MRI of MDD patients taking antidepressants has shown changes in GMV (Dusi, Barlati, Vita, & Brambilla, 2015). Remission of depressive symptoms after medication is associated with increased GMV in the right ACC, increased functional and metabolic activity, and altered connectivity in the cingulate bundle (Korgaonkar, Williams, Song, Usherwood, & Grieve, 2014). The GMV of the bilateral ACC may be particularly sensitive to the clinical response to pharmacological treatment (Liu et al., 2017). In this study, the results were still statistically significant when first episode, medication status, and disease duration were included as covariates. Therefore, the results of the current study are robust.

## Limitations

It is worth noting that this study has several limitations. First, participants in this study were recruited from multiple clinical centers, and therefore some of the clinical data were incomplete, such as the name of the medication used, the dosage, and the parameters of the MRI scanner. Second, SI was collected through the HAMD item 3 rather than a structured SI-specific instrument. Replication based on structured SI-specific instruments is needed for this study. Third, the lack of raw data did not allow us to further analyze functional indicators, such as functional brain connectivity in the ACC region in MDD patients with and without SI. Fourth, the database did not specifically record whether each patient was diagnosed through DSM-IV or ICD-10. Therefore, it is unclear how many enrolled MDD patients were diagnosed with ICD-10 and DSM-4, respectively. This gap should be remedied in future studies by using the same criteria or by precise documentation. Finally, there was a large difference in HAMD scores between MDD patients with and without SI, thus further studies are needed to investigate the relationship between depression severity and SI and structural brain abnormalities.

## Conclusion

In conclusion, our study found that the GMV in the ACC region was significantly smaller in MDD patients compared to healthy controls. Compared to MDD patients without SI, MDD patients with SI had higher HAMD scores, greater GMV of the bilateral ACC, and significantly less atrophy. The GMV in the bilateral ACC of MDD patients was positively correlated with SI. We hypothesized that ACC may be involved in the pathological mechanism of SI in MDD patients, and GMV of ACC may be a biomarker of SI in MDD patients. However, there were methodological limitations of this study, including incomplete clinical data, no use of a specific structured SI tool, lack of raw data, and differences in HAMD scores between MDD patients with and without SI. Therefore, there is a need to assess MDD patients with small differences in HAMD scores using a specific structured SI tool, and to collect their complete clinical data and raw data to understand the mechanism of differences in GMV of ACC between MDD patients with and without SI.

## Supporting information

Yi et al. supplementary material Yi et al. supplementary material

## Data Availability

The data that support the findings of this study are available from the corresponding author, (ZXY), upon reasonable request.

## Supplementary material

The supplementary material for this article can be found at http://doi.org/10.1017/S0033291724003283.
