# Supplementary material for: Structural brain changes in the anterior cingulate cortex of major depressive disorder individuals with suicidal ideation: Evidence from the REST-meta-MDD project
Source: Psychol Med. 2025 Feb 7;55:e24. doi: 10.1017/S0033291724003283 (PMC12017364; doi:10.1017/S0033291724003283)
Supplement: Yi et al. supplementary material [file S0033291724003283sup001.docx]

**Table S1. Regions Showing Significant Difference Between GMV of MDD patients with SI and without SI after adding covariates**

| Brain areas | Cluster size | BA | L/R | MNI Coordinate of Peak Voxel | | | Tvalue  (peak) |
| --- | --- | --- | --- | --- | --- | --- | --- |
|  |  |  |  | x | y | z |  |
| Cingulate Gyrus, anterior division | 168 | 24 | L | -6 | 18 | 33 | 5.192 |
| Cingulate Gyrus, anterior division | 51 | 24 | R | 1.5 | 33 | 15 | 4.702 |
| Cingulate Gyrus, anterior division | 6 | 0 | R | 1.5 | -4.5 | 28.5 | 4.208 |
| Cingulate Gyrus, anterior division | 5 | 23 | R | 1.5 | -15 | 43.5 | 6.033 |

Note: BA= Brodmann area; MNI coordinates= Coordinates of primary peak locations in the Montreal Neurological Institute space; T-statistical value of peak voxel showing GMV differences between groups; 1000 permutations with TFCE corrected for multiple comparisons across space.

**Table S2. Associations between the GMV in ACC and age as well as SI in MDD patients**

| Variables | Brain areas | Cluster size | BA | L/R | MNI Coordinate | | | r | p |
| --- | --- | --- | --- | --- | --- | --- | --- | --- | --- |
|  |  |  |  |  | x | y | z |  |  |
| age | Cingulate Gyrus, anterior division | 158 | 24 | L | -6 | 31.5 | 18 | -0.337 | <0.05 |
|  | Cingulate Gyrus, anterior division | 94 | 24 | R | 4.5 | 36 | 7.5 | -0.295 | <0.05 |
|  | Cingulate Gyrus, anterior division | 60 | 0 | R | 4.5 | 24 | 16.5 | -0.284 | <0.05 |
|  | Cingulate Gyrus, anterior division | 37 | 11 | L | -3 | 37.5 | -6 | -0.310 | <0.05 |
| SI | Cingulate Gyrus, anterior division | 148 | 24 | R | 1.5 | 15 | 28.5 | 0.367 | <0.05 |
|  | Cingulate Gyrus, anterior division | 105 | 24 | L | -6 | 18 | 33 | 0.323 | <0.05 |
|  | Cingulate Gyrus, anterior division | 28 | 23 | R | 1.5 | -15 | 28.5 | 0.337 | <0.05 |

Note: SI= suicidal ideation; BA= Brodmann area; MNI coordinates= Coordinates of primary peak locations in the Montreal Neurological Institute space; P-value for pearson correlation; Adjusted by GRF, cluster-P < 0.05, voxel-P < 0.001.


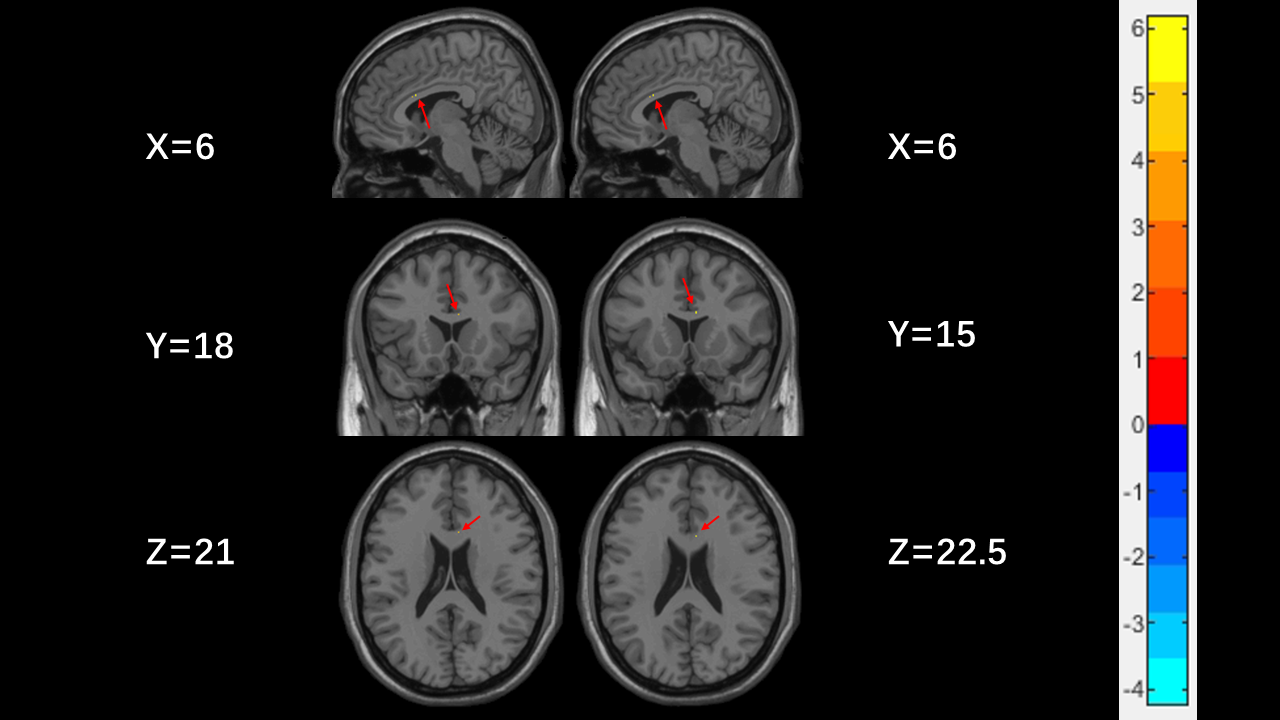


**Figure S1.** Clusters with significant differences in GMV in ACC between MDD patients and healthy controls based on two-sample t-tests. Red and blue colors denote increased and decreased GMV. The red arrow shows the corresponding brain region. The color bars indicate the T-value (1000 permutations with TFCE correction).


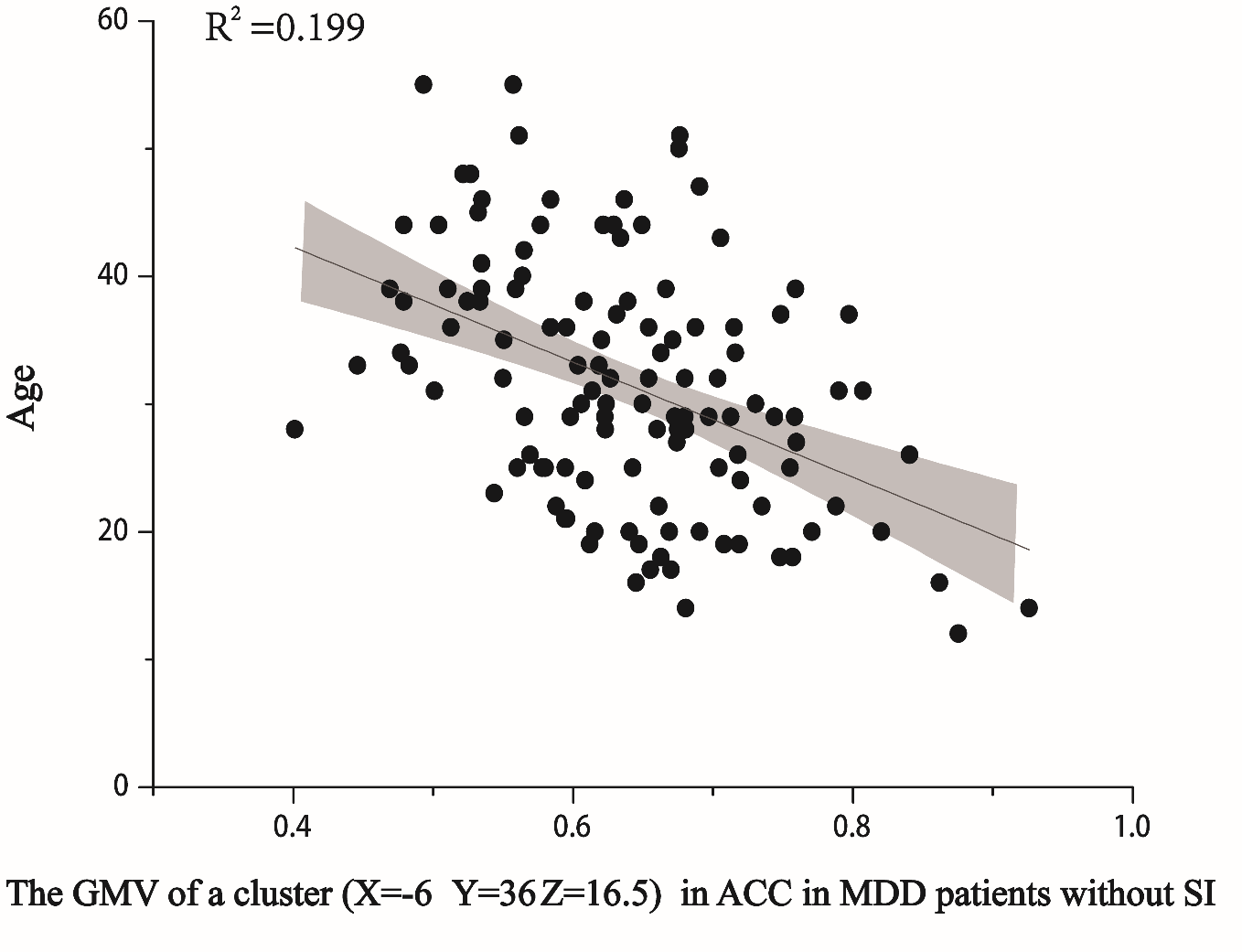


**Figure S2.** Significant negative correlation between GMV of a cluster (X=-6 Y=31.5 Z=18) in ACC and age in MDD patients without SI (p < 0.05).
